# Challenges, strategies and consequences from the perspective of German nursing home managers during the first wave of the COVID-19 pandemic - a qualitative interview study

**DOI:** 10.1186/s12877-023-03787-4

**Published:** 2023-03-23

**Authors:** Marco Sander, Richard Dano, Anja Bieber, Anna Dammermann, Steffen Fleischer, Claudia Dinand, Martin Müller, Ralph Möhler, Kristin Schultes, Sascha Köpke, Martin N. Dichter, on behalf of the HEICO-study group, Swantje Seismann-Petersen, Daniel Matthies, Sabine Sommerlatte, Gabriele Meyer, Linda Steyer, Sebastian Isensee, Katrin Balzer, Margareta Halek, Stefanie Freytag, Ilona Hrudey, Claudia Hasenpusch, Astrid Eich-Krohm, Sarah Meyer, Alexandra Piotrowski, Falk Hoffmann, Alexander Fassmer

**Affiliations:** 1 Institute of Nursing Science, University of Cologne, Medical Faculty and University Hospital Cologne, Cologne, Germany; 2 Institute for Health and Nursing Science, Medical Faculty, Martin Luther University Halle-Wittenberg, Halle (Saale), Germany; 3 Institute of Social Medicine and Epidemiology, Nursing Research Unit, University of Lübeck, Lübeck, Germany; 4 Department of Nursing Science, Faculty of Health, Witten/Herdecke University, Witten, Germany; 5 Faculty of Applied Health and Social Sciences, Rosenheim Technical University of Applied Sciences, Rosenheim, Germany; 6 Department of Primary Care and Health Services Research, Medical Faculty Heidelberg, Heidelberg University, Heidelberg, Germany; 7 Institute for Health Services Research and Health Economics, Centre for Health and Society, Medical Faculty and University Hospital Düsseldorf, Heinrich-Heine-University Düsseldorf, Düsseldorf, Germany; 8 Public Health Centre, Department Health Sciences, University of Applied Science, Fulda, Germany

**Keywords:** COVID-19, Nursing homes, Qualitative study, Challenges, Strategies, Consequences

## Abstract

**Background:**

The first wave of the COVID-19 pandemic reached Germany between March and May 2020. In order to contain the spread of the virus and particularly protect vulnerable people, the government imposed a lockdown in March 2020. In addition to infection control measures, such as hygiene and social distancing requirements, a general ban on access to nursing homes for relatives and external service providers was issued.

**Methods:**

To investigate the challenges and consequences of the enacted infection prevention measures and specific strategies for nursing homes in Germany, a multicentre cross-sectional qualitative interview study with nursing home managers and ward managers was conducted. Recorded audio data were transcribed, analysed using thematic framework analysis and reflected in peer debriefings.

**Results:**

Seventy-eight interviews with 40 nursing home managers and 38 ward managers from 43 German nursing homes were conducted. At organisational level, the following six themes were identified: Appointing a multi-professional crisis task force, reorganizing the use of building and spatial structures, continuous adaption and implementation of hygiene plans, adapting staff deployment to dynamically changing demands, managing additional communicative demands and relying on and resorting to informal networks. To deal with the pandemic challenges also six themes can be described for the direct care level: Changed routines, taking over non-nursing tasks, increased medical responsibility, increased documentation demands, promoting social participation and increased communication demands. Also various negative consequences were identified (four themes): Psychological stress, negative emotional consequences, permanent feeling of responsibility and increased potential for conflicts. Positive emotional consequences were also reported (two themes): resources for the challenges and positive emotional consequences for home managers and staff.

**Conclusions:**

The results of the described challenges, strategies and consequences allow recommendations as basis for possible approaches and successful adaptation processes in nursing home care in the future. In particular, there is a need for local networks to act in a coordinated way and a need for quantitative and qualitative support for nurses, such as staff support as well as advanced nursing practice, to cope with the challenges of the pandemic.

## Background

The SARS-CoV-2 virus has caused a global pandemic that has proceeded in several waves and is still ongoing. In Germany, the first case of infection was diagnosed at the end of January 2020 [1] and the first wave of the infection hit Germany from March to May 2020 [2]. People living in nursing homes were particularly affected by the consequences of the pandemic. During the first wave, as in other European countries, COVID-19 death rates were particularly high among nursing home residents in Germany compared to the total population [3]. Nursing home residents are at high risk of a COVID-19 infection because of their vulnerability (e.g. age-specific pathophysiological changes, reduced performance of the immune system) and the potentially increased transmission of pathogens, for example due to shared provision of food, group activities and multiple contact with nursing home staff [4]. Also, residents have an increased risk for severe courses of the disease and higher mortality rates associated with COVID-19 [5, 6]. During the first COVID-19 wave [7], this led to strict application of infection control measures in many countries, including no-visiting- and room–isolation-policies in nursing homes. In addition, many social and group activities were restricted in favour of infection control, e.g. activities to promote exercise, cognitive stimulation or shared meals. These restrictions resulted in reduced staff-resident contact time, lower levels of physical activity, and reduced social interactions. To enable a minimum level of social participation, different contact minimising visiting procedures (e.g. behind transparent barriers or windows) [8] or communication via telephone and video calls were established [9].

At the same time, the pandemic posed an extraordinary challenge for nursing home managers. For example, support and protection of staff as well as their scheduling and cohorting were important challenges. For Germany, it should be noted that, especially in the first wave of the pandemic, no evaluated recommendations for dealing with the pandemic were available. There were federal state-specific guidelines for infection control, which were frequently adapted in the course of the pandemic [10]. However, specific implementation and handling of the consequences for individual residents were regarded as responsibility of facilities. Thus, each institution developed individual solutions for the existing challenges. To explore this situation, the multi-centre and multi-professional study group “Nursing homes in the COVID-19 pandemic” (German acronym: HEICO), a network of ten, predominantly nursing research institutes throughout Germany, investigated two major research questions that could be applied as a basis for the identification of best practice solutions for handling the challenges posed by the pandemic.

1. What challenges, strategies and consequences have emerged due to the COVID-19 pandemic for the organisational level in German nursing homes?
2. What challenges, strategies and consequences have emerged due to the COVID-19 pandemic for the direct care level in German nursing homes?

## Methods

### Design

This multicentre study used a qualitative descriptive design following the criteria for reporting qualitative research (COREQ) [11]. Data collection was based on semi-structured telephone interviews with nursing home managers and ward managers from 43 nursing homes. Recruitment and data collection was carried out within the HEICO consortium. The ethics committee of the Martin-Luther University Halle-Wittenberg (number 2019–006 of 25.05.2020) approved this study. Interviews as well as data analyses were based on Standard Operation Procedures for recruitment, interview conduct, data management and analysis, with the aim of ensuring comparable procedures.

### Participants and recruitment

A convenience sample of nursing homes was recruited in various regions throughout Germany from April to May 2020 based on established co-operations between study centres and nursing homes. During the recruitment process, attention was paid to the heterogeneity of the institutions in terms of regional settlement, provider and size of nursing homes. Nursing home managers and ward managers were invited to participate either via E-mail or telephone. Eligible were nursing home managers and ward managers who were regularly on duty in their facilities during the first wave of the pandemic. Ward managers had to have a qualification as registered nurse.

The response rate of the invited nursing homes that could be reached for a short verbal information was 100% due to the established cooperation between study centres and nursing homes. Nursing home and ward managers received verbal and written information about the study. After obtaining written informed consent, an appointment was made for a telephone interview. Recruitment and interviewing took place in parallel. Recruitment was stopped when the interviews yielded no new information (saturation of data).

### Data collection

Interviews took place during and directly after the first wave of the COVID-19 pandemic in Germany from May until the end of July 2020. Telephone interviews were based on two different semi structured interview guides, one for nursing home managers and one for ward managers. Interview guides were developed based on the research questions and discussions by the study group before data collection. Interviews were audio recorded and transcribed according to the rules of Dresing and Pehl [12]. The interviewer was consulted in case of uncertainties within transcription process. Descriptive data of nursing homes and interviewees were collected prior to the interview using a structured data collection sheet.

### Data analysis

The analysis was based on the thematic framework analysis [13] using the software MAXQDA 18 [14]. This analysis method allows analyses of predefined categories and also identification of new categories in the data [15].

The analysis was conducted based on the following steps:

1. *Familiarization:* First abstraction and conceptualization based on the transcription of the interviews
2. *Identifying a thematic framework:* Creation of a framework, abstraction and conceptualization along the research questions and the interview guide (Table 1 shows the thematic framework as result of step 2)
3. *Indexing:* Coding of the data according to the topic matrix
4. *Charting:* Thematic summary, with verification of indexing
5. *Mapping and interpretation:* Systematic recognition process: interrelationships have been worked out and concepts defined

**Table 1:** Thematic framework

No	Codes
1	Description of the situation
2	Changes due to the specifications
3	Implementation of the requirements for cohorting/isolation
4	Effects of contact restriction on daily activities
5	Measures to promote social participation and quality of life
6	Interaction + communication regarding the measures
7	Changed effort due to pandemic
8	Emotional consequences
9	(Current) challenges posed by the pandemic
10	Support needs
11	Personal protective equipment and disinfectants
12	Demands on politics
13	Public image/esteem
14	Consequences
15	Responsibilities

The first step was conducted by at least one researcher per study centre, steps two to four were conducted by a core working group of five researcher. This core working group met regularly during the analysing process to reflect data analysis procedures. The fifth step was performed by the core working group and reflected by all authors. After using the initial thematic framework from step two to code the data, new categories were found in each step of analysis, leading to the final themes of our study.

## Results

### Participants

Most nursing homes were run by welfare organisations; the mean number of beds was 96.8 (± 33.9) (Table 2). In total, 40 nursing home managers and 38 ward managers from 43 nursing homes participated. Participants’ characteristics are presented in Table 2. As sometimes one nursing home manager was responsible for two homes, the number of nursing homes does not correspond to the number of managers. For five nursing homes, no interviews with ward managers were conducted. Interview durations ranged from 20 to 101 min with a mean length of 49.8 min (± 19.1).

**Table 2:** Characteristics of the sample

	**Nursing home managers** **(** ***n*** ** = 40)**	**Ward managers** **(** ***n*** ** = 38)**
Work experience in years	22.4 (± 9.6)	20.0 (± 10.1)
Work experience in current function in years	10.2 (± 8.9)	8.9 (± 6.9)
Experience with infectious diseases that required advanced measures	40 (100)	37 (97.4)
Nursing home (*n* = 43)		
Type of nursing home provider		
Public	2 (4.6)	
Welfare	30 (69.8)	
Private	11 (25.6)	
Number of beds per nursing home	96.8 (± 33.9)	
Nursing homes with specific nursing focus according to the care contract with the statutory long-term care insurance		
Dementia	8 (18.6)	
Gerontopsychiatry	1 (2.3)	
Mental disabilities	1 (2.3)	
Mechanical ventilation	1 (2.3)	
Persistent vegetative state	1 (2.3)	
Others	5 (11.6)	
Nursing homes with confirmed COVID-19 infections cases among residents		
No	33 (76.7)	
Yes	7 (16.3)	
Not reported	3 (7.0)	
Number of infections per nursing home	6.1 (± 15.9)	
Nursing homes with confirmed COVID-19 deaths among residents		
No	34 (79.1)	
Yes	6 (13.9)	
Not reported	3 (7.0)	
Number of deaths per nursing home	1.3 (± 3.7)	
Nursing homes with confirmed COVID-19 infections cases among staff		
No	27 (62.8)	
Yes	13 (30.2)	
Not reported	3 (7.0)	
Number of infections per nursing home	2.3 (± 5.9)	

Values are numbers (%) or means (± SD)

### Themes for organisational and direct care levels

The thematic analysis resulted in six themes for the organisational level and six for the direct care level. Results include strategies for implementing measures taken and resulting consequences in nursing homes, which are presented following the results for both levels. Challenges are indirectly described through the identification of these strategies.

### Challenges and strategies for the organisational level

**Fig. 1:**
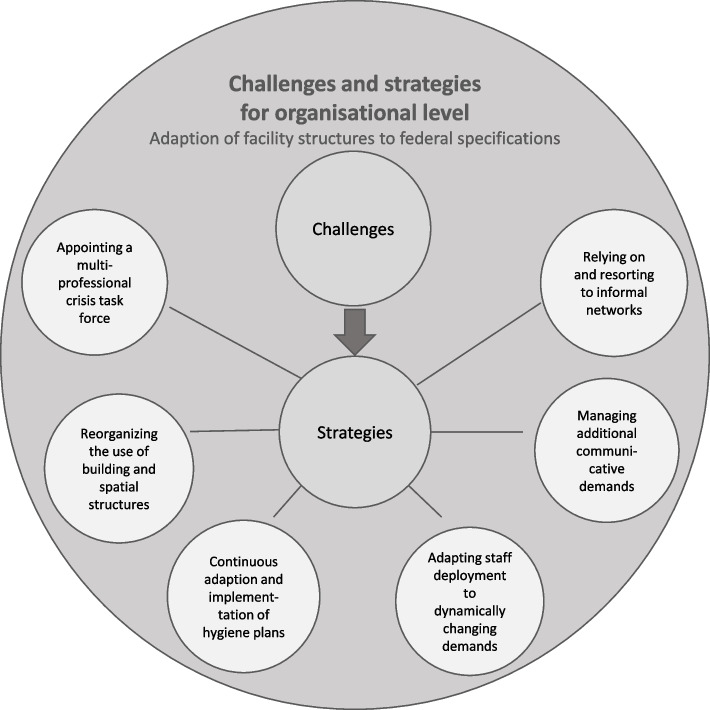
Challenges and strategies for organisational level here

COVID-19 led to sudden and unforeseen challenges for nursing homes on the organisational level, e.g. adapting federal requirements to local conditions or ensuring infection prevention with limited resources. Consequences, mirrored in increased potential for conflicts and enhanced responsibilities for nurses, had to be managed applying different measures and strategies. Especially consequences of challenges for both levels are mutually depending. Based on the analysis, six topics for the organizational level could be identified. Results for the organisational level are summarised in Fig. 1.

#### Theme 1: Appointing a multi-professional crisis task force

Most of the nursing homes formed a team involving members of nursing homes’ quality managers, hygiene specialists and safety officers in order to create and develop a pandemic concept. The unforeseen and exceptional COVID-19 situation required an extended workload for the multi-professional crisis task force. In addition, team members were sometimes permanently on call in order to apply recurring changes in the prevention plan regarding federal orders. Employees had to be informed about every step and changes of the pandemic concept.

“And we meet at least twice a week, when new guidelines or regulations come in, when there is something new, then spontaneously more often and really, it has really proven itself. In the meantime, it has become a really great group, the facility management is there, the housekeeping management, me as the nursing service manager and hygiene officer, and we also sometimes include ward management when something is going on on the ward and needs to be discussed or clarified. And that makes perfect sense to sit down together.” (02_EL_05)

#### Theme 2: Reorganizing the use of building and spatial structures

To ensure minimal physical contact for residents and staff members the usual access processes into the nursing homes were changed. Employees were assigned to use distributed entrances to avoid an accumulation of persons.

Furthermore, most nursing home managers applied a strict separation of living areas to avoid exchange between different wards and supported social distancing. Fixed allocation of seats in dining areas were applied to minimize contact between residents and staff. In case of suspected or confirmed COVID-19 infections, additional rooms equipped with isolation materials were used for quarantine purposes. The establishment of specially created visiting rooms gave relatives the possibility to visit residents under restricted circumstances (e.g. plexiglas panels between residents and visitors, distancing, documentation of personal data). However, relatives were allowed only limited visiting times and had to comply with hygiene regulations.

“We thought about it for a very long time because we had to create infection areas and quarantine areas. That we have to turn double rooms into triple rooms, single rooms into double rooms. Because how else are you supposed to separate the areas?! Because we are not a hospital. We don't have any locks, do we? We don't have any extra rooms and that was a real challenge for us.” (06_WBL_07)

#### Theme 3: Continuous adaption and implementation of hygiene plans

Already existing hygiene plans had to be extended and continuously adapted with an ongoing intensification of specific measures. While hygiene plans had previously been geared towards dealing with other infections e.g. norovirus, they now had to be extended to include hygiene rules for avoiding COVID-19 infections. This meant intensified hand disinfection, wearing face masks, social distancing, creating isolation rooms as well as increased disinfection of everyday objects.

"Basically, these are measures that we all know and can carry out, that we have all standardised, and that we now only have to apply permanently, in other words, this specialised hygiene has now become basic hygiene for us.” (02_EL_05)

A major challenge with these measures was the difficult organisation of material procurement due to general and nationwide deficiencies of protective equipment in the early phase of the first wave, e.g. regarding disinfection sprays, masks, gloves etc. In some cases, strategies included activating personal networks like local distilleries or fire brigades. In addition, specific and needed material led to increased costs.

#### Theme 4: Adapting staff deployment to dynamically changing demands

One of the biggest challenges for the organisational level was the increased need for staff due to additional workload. Employees had to cover tasks in order to meet the demands of new infection control measures as well as to ensure patient centred care. This included permanent control of contact restrictions, increased effort due to enhanced hygiene regulations and handling needs of relatives. Relatives needed comprehensive information about implemented infection control measures and strategies were sought for them to communicate with their relatives. In most nursing homes, it was necessary to reschedule staff, for example, when some employees had a COVID-19 infection or needed to go into quarantine.

There was a need to increase night staff to meet the challenges of expanded workload during the night. Another strategy was the involvement of employees from temporarily closed services like day care services, restaurants or cafeterias. Staff members were assigned to support staff in residential care e.g. concerning the implementation of infection control measures (e.g. organisation of visits to relatives). Loan workers were another strategy to increase staff capacity. However, in some cases, nursing homes deliberately decided against recruitment of loan workers in order to keep staff total numbers at a minimum and thereby reducing the risk of introducing infection through staff.

Nursing home managers also considered additional burden for regular nursing staff because of a necessary familiarization of external staff.

“[…] now in such a crisis, to fall back on outside employees, to familiarize them with the processes, um, I think is quite difficult." (09_EL_02)

Reorganisation of the deployment of nursing students resulted in lack of educational nursing input for students as the focus of their work in nursing homes was changed to support regular staff and take over care tasks (serving food, grocery shopping, accompanying residents to activities).

Some nursing homes prepared themselves by creating hypothetical duty rosters in the event of a possible outbreak of COVID-19. There were no special features in annual leave planning and holiday bans were only partially used.

#### Theme 5: Managing additional communicative demands

The ban on visits for relatives and external service providers hindered communication and contact for nursing home managers.

During the first lockdown in Germany relatives, friends, and legal guardians were not allowed to have any personal contact with residents. Nursing homes therefore introduced several technical communication forms. Some facilities arranged video calls in order to support visual communication and therefore create a livelier conversation with relatives, more similar to a real-life talk. In most cases, nursing home managers took over the organisation or delegated these tasks to staff members. Some nursing homes acquired digital equipment such as notebooks, smartphones or tablets to enable video-based calls, usually accompanied by staff to help with technical problems. According to the interviewees the most relevant fact was that the organisational level as well as nursing staff had to take extra time in order to instruct residents to use the electronic devices.

“[…] each living area got a tablet, so that you can skype and […] make appointments also with relatives […]. That is very well received. Where the grandchildren can then also contact the grandparents. We also have received additional smartphones that can be called and WhatsApp to send photos back and forth.” (06_EL_02)

Communication and interaction between nursing home managers and external service providers also increased. During the lockdown, physiotherapists, physicians, podiatrists and pharmacists for example regularly had to cancel personal visits. Consequently, therapies were reduced to a necessary minimum or covered by nursing staff in order to maintain preventive measures.

Managers acted as gatekeepers in terms of communication and organisation, e.g. passing on important messages from external service providers to nursing staff. The absence of direct communication offered room for misconceptions and misunderstandings and led to more communication need.

"But really the flow of information about changes, changes in laws and regulations, that was quite, quite annoying, quite difficult.” (04_EL_02)

#### Theme 6: Relying on and resorting to informal networks

The pandemic brought challenges that had been unknown and required unusual actions. Nursing homes particularly used informal networks for the acquisition of protection material (masks, gloves, disinfection liquid). Personal contacts of staff or managers offered support and showed solidarity. Numerous friends and colleagues sewed masks and helped organising materials.

“We [asked] a social support worker who sat down at the sewing machine and sewed masks.” (05_WBL_03)

“So there’s a distillery near us, they made disinfectant liquids there.” (07_WBL_03)

To ensure residents’ quality of life and to offer diversity in daily activities, nursing homes reached out to schools and kindergartens. Online presentations and meetings were held where teachers organised choir and play performances for entertainment. In some cases, school and kindergarten children performed in front of the nursing home buildings and residents enjoyed the singing and plays at their windows or from their balconies.

### Challenges and strategies for the direct care level

The results for the direct care level are influenced by the results of coping with the situation at the organisational level and consequences are mutually dependent. Challenges for direct care were the adaption of facility structures to infection control measures like the general ban on visits by relatives.

**Fig. 2:**
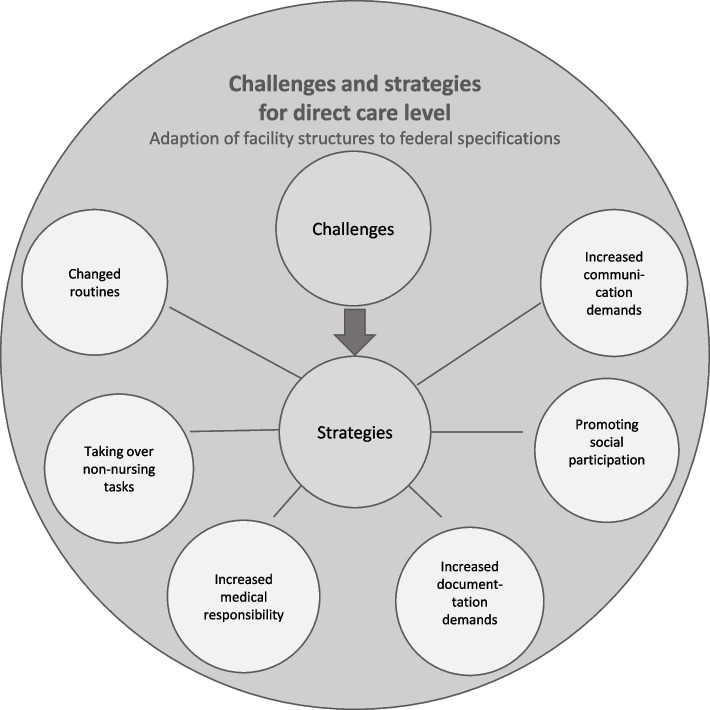
Challenges and strategies for direct care level

Comparable to results for the organisational level, data also showed six themes of challenges for the direct care level due to the initial situation in the COVID-19 pandemic (Fig. 2).

#### Theme 1: Changed routines

Due to serious changes of regular work routines, daily routines were confusing for both residents and staff. Infection control measures meant that it was no longer possible for residents to eat together. The implementation of food distribution and support with food intake in individual residents’ rooms required more time and staff deployment than usual. It was also observable that residents ate less, because of the unexpected and sudden changes of procedures.

"For example, the room service was very time-consuming. That's four meals a day and then in every room, with setting the table, covering the table, bringing it to the next room when requests are made.” (03_EL_04)

Also psychosocial support, such as cognitive stimulation or physical exercises, could no longer take place in groups, but on individual levels to control infections. This meant, for example, that not all residents were able to choose and the number and variety of offers had to be reduced. In some cases, residents enjoyed getting an individual social accompaniment. However, this sometimes led to further social withdrawal with a tendency towards depressive mood on residents’ levels.

Especially the care and treatment in quarantine or isolation rooms of COVID-19 infected residents or in case of infection suspicion led to additional planning effort with changed routines, increased staff needs and use of movement restrictions.

#### Theme 2: Taking over non-nursing tasks

Another challenge for nursing staff was the necessity to take over non-nursing tasks, which had to be organised and carried out by nurses due to the general ban on external service provider visits. These tasks included accompanying visits and later also carrying out COVID-19 tests on residents and relatives.

"Now, for example, with the visitors, we have to get them in, we have to keep exact records and check again how long they are in the rooms. That's all, it takes an insane amount of time and now you realise that they [nursing staff] can't do it anymore." (04_EL_02)

Furthermore, nurses had to take over the external service providers’ tasks, for example chiropody, hairdressing, physical exercises or household chores like disinfecting residents´ rooms. Another challenge was to instruct relatives and visitors in implementing hygiene measures and restrictive contacts with residents. The necessity of accurate control as well as a higher effort of communication with relatives caused additional burden and workload for staff.

#### Theme 3: Increased medical responsibility

Another challenge matter was the huge increase in medical responsibility for nurses, who had to deal with less support as well as reduced or no visits to nursing homes by physicians and nurses had to act on their own responsibility. Some interviewees reported nursing staff taking critical and medical decisions (e.g. order or discontinue medication) out of desperation and lack of support.

Also, avoidance of hospital admissions to reduce risk of infection for residents led to increased decision-making in critical situations by nursing staff that are usually physicians’ tasks e.g. in case of unclear pain, deterioration of residents’ general condition or suspected signs of infection. Nurses regularly felt to exceed their competence, to act outside legal regulations and to lack support by medical staff. In critical situations, nurses often felt let down by physicians. By observing acute deteriorations of residents’ health, nurses often found themselves in a dilemma: actively deciding on medical treatment (e.g. prescribing or changing medication), while trying not to exceed their own competences by taking medical responsibilities which are legally prohibited. Nurses reported these experiences as most challenging with a need for more staff support, but also for enhanced skills reflecting advanced nursing practice, which is not regularly implemented in Germany.

"And with the physicians, the communication, that was also there in principle, so they then, that now also came more from the physician´s side, they then said, so really only if something is very urgent, we now come to the nursing home. Otherwise, everything was clarified by telephone, or, if it was urgent, i.e. something where the physician said, here, immediately admitted to the hospital, the people, right?" (07_WBL_03)

#### Theme 4: Increased documentation demands

Changed routines, increased medical responsibility and taking over non-nursing tasks were first priorities in taking measures to prevent COVID-19. Thus, documentation was in many cases regarded as negligible task that could be done at a later time point and required additional mental and concentration effort due to the uncertain situation. The amount of documentation generally increased. In order to ensure protective measures and detect infections early, vital signs and potential symptoms were constantly recorded.

"Yes, we had to record, for example, in the documentation, we had to measure the vital signs twice a day for a fortnight, temperature, blood pressure, pulse, breathing rate, oxygen content in the blood. That also had to be documented, that is already an additional effort for a complete residential group with 17 residents, to do that twice a day and to document it accordingly." (06_WBL_02)

Documentation was also necessary regarding the access and exit of visitors in order to record individual contacts and detect potential viral carriers in case of a COVID-19 outbreak. Staff members, residents and visitors had to follow general hygiene plans, which also had to be documented daily.

#### Theme 5: Promoting social participation

To ensure social participation of residents due to the general visit ban for relatives and external service providers, staff had to create services for residents to prevent isolation and boredom. One of the most often used strategies was accompanied letter writing and reading aloud letters by relatives or other cooperating institutions like local kindergartens or schools. Also, there was an increased offer of accompanied individual walks through the neighbourhood. Relatives often used visits at fences or balconies. Events like music performance took place in front of nursing homes or in the gardens where the residents would be able to watch from their rooms. Nurses had to accompany or organise these activities. In some nursing homes, nurses recorded and presented church services or video messages by pastoral workers. One nursing home offered physiotherapy via video conferencing, but the costs for this offer were not refinanced by the health insurance, so this offer had to be cancelled.

"But yes, we all tried to bridge the gap somehow or to sit down in the [residents´] room and read a bit or do something." (05_WBL_03)

#### Theme 6: Increased communication demands

The overall pandemic situation led to a need of increased individual conversations with residents and their relatives. The perception of the situation was described as frightening and menacing. In order to reduce fears in relation to the loss of loved ones or one's own death, nursing staff made additional efforts to provide emotional support to residents and relatives. Nurses considered this sort of communication as their duty to ensure residents’ wellbeing.

In general, communication was difficult during the first lockdown. The necessity to wear protective equipment (e.g. masks) during the whole day led to challenges in communication and interaction with cognitively impaired residents. Difficulties in interpreting facial expressions and provoking confusion were results of a hindered communication.

"Yes, you can definitely tell by the eyes if someone is smiling or if they are in a bad mood, but for the residents it was hard to tell [through the masks]. And it's still the case that they have to ask several times because many people look at the mouth and can understand by seeing and hearing what we want to do with the resident. So that is difficult." (03_WBL_03)

Moreover, infection control measures resulted in a changed handover structure. For example, most institutions performed their handover with one nurse per shift, which led to additional communication efforts as information about residents had to be transmitted to other staff on the shift. Also, this resulted in information gaps.

### Supporting factors for the work at organisational and direct care levels

The challenges of the pandemic situation were time consuming and required extended organisational skills from the management level. Despite the fact that normal life had to be reorganised, nursing home managers endeavoured to maintain appropriate work and life procedures. Interview participants reported an enormous workload. Nevertheless, well-functioning cooperation with relatives and external service providers in combination with high motivation of nursing home teams helped to maintain professional standards, which was often supported by a pronounced sense of solidarity and increased attentiveness of staff and leaders.

“Quite simply, being there for each other is important and as long as you support each other […] because if that's not the case, then it's very difficult, of course. […] But as long as the cooperation is as good as it has been so far, that people support each other, I don't know what I could say as support. We just hope that this time will also be over at some point.” (06_WBL_04)

### Consequences of the challenges and strategies in nursing homes

Challenges with the adaption of facility structures and processes to infection control measures and resulting strategies led to several consequences for both the organisation and the direct care level.

### Positive consequences for nursing home managers and staff

#### Theme 1: Resources for the challenges

COVID-related challenges were accompanied by some unexpected supporting resources. Especially the support of the team by nursing home and ward managers who were actively involved in the care of residents in some institutions was mentioned. Regularly, postponed quality checks by federal institutions and care level classification of residents by health insurances were considered as a relief especially as nurses and managers were relieved of the need to accompany these visits and the related preparation and documentation.

"…that was of course quite exciting, but the first time I must say, when no one came any more, no visitors either, and that was a nice time, you could work off a lot of things that were left lying around, because there were few calls and also no quality audits by the Medical Service of the health insurance, no care classification in that sense." (01_EL_03)

#### Theme 2: Positive emotional consequences for nursing home managers and staff

Especially a feeling of a newly established team cohesion was reported by nearly all participants, which can be seen as a resource to deal with the challenges.

"…so among ourselves I would say that we have actually coped well and have actually strengthened ourselves as a team, (…) I would say that we have also strengthened ourselves a little bit." (07_WBL_03)

Most interviewed ward managers reported a feeling of a calm atmosphere at the residents’ level due to the absence of external offers and visits and within the possibility to provide a more holistic care.

"We might have a bit more time to go through the rooms. Because the whole work situation is a bit quieter. No contact from the outside. That is something different. You can deal with the people there in a different way." (05_WBL_03)

In addition to nursing home managers’ reported feeling of a newly established team cohesion, nurses realised a stronger sense of connection between nursing staff and residents.

"Because especially here in the area of dementia, everything is about touching, guiding, closeness, physical closeness. Even taking people who are crying in your arms and yes, you can't always keep the safe distance of 1.5 m and that affects you.” (06_WBL_02)

The overall positive emotional consequences for managers and nursing staff turned into resources for all employees in nursing homes. Despite many challenging situations, staff regularly detected solutions to cope with strenuous circumstances.

### Negative consequences for nursing home managers and staff

#### Theme 3: Psychological stress for all facility employees

Participants described an increased stress level among nursing home staff during the first wave of COVID-19 and therefore more potential for conflicts, especially with cognitively impaired residents and also with relatives, particularly when explaining and defending the imposed restrictions and their implementation. Nursing homes’ compliance with infection control requirements led to a huge need for clarifying discussions, mostly addressed by managers, but in some cases, especially on the phone, by nurses.

"Or, for example, relatives say: ´I don't know anyone who died of COVID-19´ or ´how high is the danger, since there are so many infected people now, that something will be brought in here? You staff can also carry it in.´ For example. That's also hard for the staff to bear and for me too." (06_EL_04)

Beside these challenges, nurses’ increased medical responsibility led to a feeling of too much responsibility accompanied by the fear of serious errors in medical care.

Most participants criticised the lack of transparency and accuracy in information transmission by the federal government as well as the organisation and coordination of infection control measures by the authorities, which led to a feeling of uncertainty. Because of this, participants demanded more information and safety.

#### Theme 4: Negative emotional consequences

Negative feelings were reported to be caused by perceived lack of support from federal institutions and regulatory bodies as well as lack of protective equipment, resulting in feelings of anxiety and financial, psychological and work-related stress. Here, one important aspect was the lack of experience with such a widespread pandemic situation. Mostly, increases in medical responsibility and potentials for conflicts were perceived to lead to a permanent feeling of psychological stress.

"Well, that's a bit shocking then. No? That you get NOTHING from the doctors and no real information from the health authorities.” (07_EL_03)

The burden of COVID-19-related deaths was only mentioned in one interview. The interview participant described the pandemic as very stressful for everyone involved in the entire facility, especially since there was a prolonged period with several deaths. After the first death among residents, the nursing home received support from local authorities, but it was not considered helpful.

#### Theme 5: Permanent feeling of responsibility

The challenges concerning altered and increased responsibility during the pandemic were perceived as burdensome emotional factors. Nursing home managers had to work extra hours, which resulted in a feeling of helplessness and perception of lack of support from the government or other institutions.

"But I have never been or felt so in the forefront and in charge, with all the consequences." (06_EL_02)

Ward managers reported a feeling of fear because of the lack of protective equipment and the uncertainty without prior experiences, leading to a feeling of helplessness. Although most nurses had an understanding of federal guidelines, they also had to deal with a lack of clear instructions. They also reported a permanent feeling of responsibility, especially for their residents’ wellbeing and health and for themselves and their families. Especially in case of COVID-19 infections in their personal vicinity, nurses had to deal with a feeling of threatening residents.

"But to have this feeling, that is what ALL staff members said, that they are somehow afraid of getting the virus and maybe infecting some resident. (…) To have the guilt, maybe I am the one who is to blame, that the residents are tested, that quarantine comes, that everything is closed again. That always floats along." (06_WBL_07)

In comparison to the structures in hospitals, nursing staff felt a sense of injustice related to their setting, in particular concerning medical attention, availability of protective equipment, and lack of testing possibilities.

"In normal times, you would say that this [inadequate medical care and lack of availability of protective equipment] is failure to render assistance." (05_WBL_03) 

In German nursing homes physicians come regularly to medical visits but are not employed in them. In the pandemic the visits were also reduced by them. Especially reduced medical care for residents led to negative feelings with a feeling of a huge responsibility for residents care as physicians avoided hospital admissions and sometimes only carried out visits by telephone.

"As I said, medical care and also overall care has of course deteriorated massively for the residents." (03_EL_01)

#### Theme 6: Increased potential for conflict

Especially the avoidance of contacts and the need for social distancing led to various challenges in non-personal communication, which was mostly conducted via phone or video-conferencing, if facility-specific allowed, which was not regularly the case.

"Of course we also had a few relatives, we also have rooms here in the basement or behind the house. The ground floor is also at ground level. They simply came in via the terrace. Yes. Again and again they were lectured and spoken to: We're not allowed to and in general. Yes, but my mother lives on the ground floor. I could go and look for her. Yes. So that was a bit of what made everything difficult. You always had to look out: Now there's another visitor. How does that work? Yes. Unreasonable relatives. That was actually the worst thing." (05_EL_02)

Nurses expressed a huge need for lobbying and more appreciation of the nursing profession.

"We just don't have a lobby. And THAT'S so sad and THAT'S what we're seeing again in the crisis." (07_EL_03)

"This networking of the entire inpatient care facilities has been lacking, that has to be said, and I think it was also a topic in the media once and there was this headline ´Care must finally deliver´, where we said: yes, we deliver all the time." (02_EL_05)

**Fig. 3:**
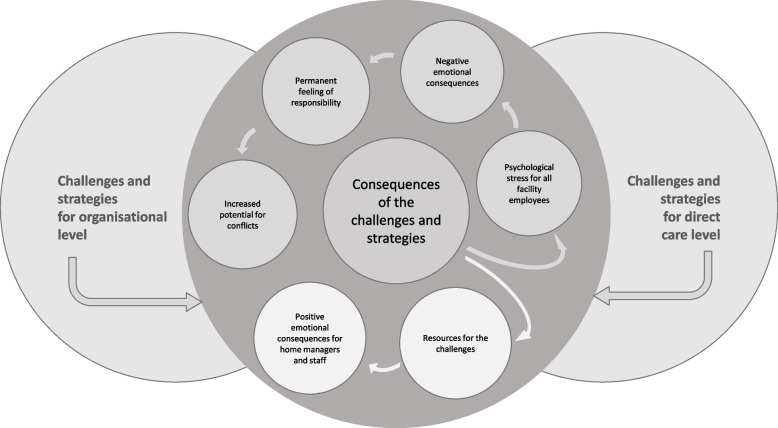
Consequences of the challenges and strategies

All reported challenges, strategies and consequences led to an overarching assessment of the overall pandemic situation as felt in spring 2020. In summary, both organisational and direct care levels are interdependent (Fig. 3).

### After easing restrictions

After the first wave of the COVID-19 pandemic a habituation effect for staff as well as for residents and their relatives was reported, especially focusing the adopted measures like wearing protective equipment and the avoidance of contacts. Nursing home managers had to remind staff to keep the prevention measures in mind. All reported an acceptance of remaining restrictions by relatives.

"I have to say that the residents have come to terms with it, I don't want to say that, but we have residents who lived through the war. And now they come and say: 'Actually, we're still doing well. We don't suffer from hunger. We're not thirsty or anything like that, and you're here too.´" (01_EL_01)

## Discussion

As in other countries, German long-term care facilities were and are particularly affected by the COVID-19 pandemic. In our multi-centre interview study with nursing home and ward managers from different regions throughout Germany, we found six themes each for challenges and strategies for organisational and direct care levels and six themes at the level of consequences (two positive and four negative).

Almost all extracted themes at organisational level are also described in the existing literature. Especially the themes *appointing a multi-professional crisis task force, reorganizing the use of building and spatial structures,* the *continuous adaption and implementation of hygiene plans* and *adapting staff deployment to dynamically changing demands* are mentioned in other studies from several countries as strategies to overcome the given challenges during the first wave of the pandemic [16–18].

The theme *managing additional communicative demands* was not directly reported in other studies, but is described as one elementary strategy to overcome other challenges, especially related to convey information about adapted federal regulations and to avoid misinformation. This might be due to the fact that nursing home managers in Germany are usually close to nursing practice and it is not uncommon for them to take over nursing duties if necessary. Other studies also report inconsistent communication about restrictions by authorities, which led to several problems and to a prioritisation of quantity instead of quality of life, which included ongoing social isolation [19]. Staff in nursing homes had to deliver care in a pandemic context and had to make existential decisions. To be informed, they used external networks; which resulted in sometimes contradicting sources of expertise and information. Moreover, the lack of prioritization by authorities led to additional work and sometimes impaired the ability of staff and managers to make decisions [20].

Also, the *relying on and resorting to informal networks* has been described in the literature in relation to adapt given restrictions by local initiatives [16]. One resource was dedicated and inventive staff and the usage of existing local networks [21]. In our results, informal networks were mostly used as a strategy to secure material procurement. In addition, the building of such local networks in nursing was described as a future strategy to overcome critical situations in Germany.

At direct care level, the themes *changed routines, taking over non-nursing tasks, promoting social participation* and *increased documentation demands* have also been described in recent publications [22, 23]. Especially people with dementia showed negative psychological and cognitive effects due to the imposed restrictions, such as increased apathy, irritability, or anxiety, which were fuelled by lack of social activities. Therefore, keeping residents to simple routines that included leaving the nursing home for exercise and stimulation should have been prioritised by staff [24], but could not be performed due to the restrictions and the increased workload. These influencing factors led to a feeling of social isolation. To avoid a general decline in psychological well-being and cognition, creative support measures for residents like increased walking, window or behind-glass visits and video calls were used, as also described in the literature [25]. To address these challenges the German Society for Nursing Science published a guideline to prevent social isolation in nursing homes under pandemic conditions [26], aiming to promote self-determination and quality of life, even under pandemic conditions.

*Increased communication demands* also concerning ongoing psychosocial individual conversations with residents and their relatives were another challenge mentioned in our interviews. Understanding necessary physical distancing measures and correct utilisation of personal protective equipment may be limited in nursing home residents, especially residents with dementia, which has been described as a potential risk to COVID-19 spread in some nursing homes [23].

During the pandemic, other complaints were sometimes neglected, trivialised or concealed by residents due to fear of inpatient hospital treatment. This was reported as *increased medical responsibility* for nurses in our study and could have led to a delayed start of diagnosis and therapy with sometimes severe consequences, which has also been reported in other studies [27]. According to one study, monitoring residents’ vital signs via telehealth was feasible, but this was not available in most nursing homes [28]. In particular, *increased medical responsibility* that comes with an increase in overall responsibility was described to contribute importantly to nurses’ workload. The scoping review of Giri et al. [16] did not report this for any other study. A likely reason for this could be that there are hardly any nurses with bachelor degrees working in German nursing homes. In addition, the comparatively unfavourable staff-to-resident ratio and the high number of untrained staff in German nursing homes supports this hypothesis. Especially the staff-to-resident ratio has been described as an influencing factor associated with excess COVID-19 transmission and mortality in nursing homes [16].

Beside the themes for challenges and strategies, we found positive and negative consequences for nursing home managers and staff. Especially the positive emotional consequences for nursing home managers and staff, like the reported calm atmosphere or the newly established team cohesion are described as a resource to overcome the situation and could be one factor to be addressed in future research.

In particular, the *permanent feeling of responsibility* led to a feeling of a too great sense of responsibility in combination with fear of serious errors in medical care, which has also been described in other studies, especially for nursing home residents with specific comorbidities [29]. The *increased potential for conflicts* due to demanding communication with residents´ relatives was another influencing factor for the perceived stress level of staff in German nursing homes. In the UK, timely, responsive, and unambiguous action according to evidence-based guidelines was described as a strategy to overcome the conflicts described [30].

Especially the work load for nurses like taking over additional work ongoing with an effort in the interaction with relatives [30] and also taking over non-nursing-tasks to avoid stress and fear for the residents were also described for Austrian Nursing homes [31]. A systematic review showed, that especially physical activities were an important topic for the residents, also prioritised and promoted by the staff during the lockdown [32]. This was not directly focused by our participants. Most of the tasks the staff took over in our study were non-nursing tasks like hairdressing, pedicure or promoting social participation, but not directly addressing physical activities. All these challenges led to psychological consequences for nurses, as fear and stress, which are described as a major effect of the pandemic [31], which were also mentioned in our interviews.

The study by Capstick et al. showed the following key-aspects*: 1) confusion and stress due to the implementation of new policies that changed very frequently, 2) the negative impact of policies such as social isolation, PPE and coronavirus testing on people with dementia and their families, 3) the impact on their own mental health and wellbeing over time and 4) creative problem solving and collaboration in the face of these challenges* [33]. These aspects are also shown in our themes. The authors also report on feelings of grief due to deaths in nursing homes. This feeling was reported only occasionally in our interviews. This may be due to the fact that the nursing homes in our sample did not show a particularly high resident mortality rate in the first wave of the pandemic. But also the lower mortality in Germany compared to other countries and at the level of nursing homes could be another reason, why this was not mentioned that often in our study [34–36].

The study by Yen et al. [37] describes the insufficient cooperation with local authorities and the inadequately enforced regulations as another challenge for nursing homes, which are also addressed in our study. But Yen et al. [37] also report about sometimes contradictory interests between protecting residents and ensuring profit in profit-oriented nursing homes, which was not shown in our analysis.

Management in particular has a central role, which can also have an impact on the stress resistance of all involved within the institutions. This requires new flexible forms of leadership [38], which have already been described in earlier theories as flexible adaption processes, for example in Fiedler's contingency model of leadership effectiveness [39] and is also shown in our results.

In summary, our results are mostly consistent with other published studies in these settings. However, there are also points, such as the influence of COVID-19-related deaths on employee’s emotions or the impact of profit orientation on the incidence of infection, which were not mentioned in our analysis.

### Strengths and limitations

Although our study is based on a comprehensive sample of qualitative date from 43 nursing homes from Germany, some limitations have to be acknowledged. First, results are limited to the first wave of the COVID-19 pandemic. Second, the convenience sample of institutions with an affinity for studies increases the possibility of answers influenced by social desirability. Another possible limitation could be researchers’ focus on relevant data material only. Therefore, we confronted the analysers with alternative interpretations through peer group reflection to establish consensual validation and intersubjectivity.

The main strength of our study is the large sample of nursing homes in different regions in Germany with a high response rate during the first wave of the pandemic. Therefore, the results are transferable and generalizable for the German healthcare system.

## Conclusion

Successful adaption processes in form of the described measures and strategies to counteract the challenges of the COVID-19 pandemic allow to develop a basis for possible approaches in the future. Especially, mutual help and support within the nursing homes but also with other institutions, specialists, experts, and professions, like physicians, in a coordinated way led to a solution-oriented and mutually supportive management of the given situation. Our results show that there is a great need for local networks to act in the described coordinated way. Another challenge and also a negative consequence which led to a feeling of permanent and insufficient responsibility and psychological stress was the inadequate medical care in German nursing homes, reinforced by the general nursing staff shortage. Furthermore, an inadequate skills-grade-mix based on the German health care system with hardly any academically trained nurses or nurses with advanced nursing skills, led to a situation with the risk of insufficient care and consequently a risk for residents´ health. Thus, there is an obvious need for quantitative and qualitative support for nurses such as staffing support as well as advanced nursing practice to deal with the challenges of the pandemic situation. Especially in view of the nursing staff shortage that existed before the pandemic and the medical responsibility in the pandemic that requires more skills and authority for nurses in German nursing homes.

Our conclusions are supported by the results of a similar study in England [21], with the difference that local networks already existed there. Another solution to overcome the described barriers could be tackled immediately through timely, responsive and unambiguous fact-based guidance [40].

Further research could focus on the longitudinal development of the described consequences for nursing home and ward managers. Also, the examination of the perceived injustice from nurses´ perspectives due to increased medical responsibility compared to nurses in hospitals should be addressed in further research, especially within the focus of the German healthcare system. Overall, a systematic summary of the existing literature regarding strategies and their effects seems warranted. Result could be used to derive commendations for future crises for nursing home management.

## Data Availability

The data (transcripts) generated and analysed during this study are not publicly available to protect participants’ confidentiality. However, they are available from the corresponding author upon personal request.
